# Targeting CCR5 as a Component of an HIV-1 Therapeutic Strategy

**DOI:** 10.3389/fimmu.2021.816515

**Published:** 2022-01-20

**Authors:** Hager Mohamed, Theodore Gurrola, Rachel Berman, Mackenzie Collins, Ilker K. Sariyer, Michael R. Nonnemacher, Brian Wigdahl

**Affiliations:** ^1^ Department of Microbiology and Immunology, Center for Molecular Virology and Translational Neuroscience, Institute for Molecular Medicine and Infectious Disease, Drexel University College of Medicine, Philadelphia, PA, United States; ^2^ Department of Microbiology, Immunology, and Inflammation, Center for Neurovirology and Gene Editing, School of Medicine, Temple University, Philadelphia, PA, United States

**Keywords:** antiretroviral drugs, CCR5Δ32, CCR5 monoclonal antibodies, CCR5 small molecule inhibitors, HIV-1 drug resistance, zinc finger nucleases, TALENs, combination therapy

## Abstract

Globally, human immunodeficiency virus type 1 (HIV-1) infection is a major health burden for which successful therapeutic options are still being investigated. Challenges facing current drugs that are part of the established life-long antiretroviral therapy (ART) include toxicity, development of drug resistant HIV-1 strains, the cost of treatment, and the inability to eradicate the provirus from infected cells. For these reasons, novel anti-HIV-1 therapeutics that can prevent or eliminate disease progression including the onset of the acquired immunodeficiency syndrome (AIDS) are needed. While development of HIV-1 vaccination has also been challenging, recent advancements demonstrate that infection of HIV-1-susceptible cells can be prevented in individuals living with HIV-1, by targeting C-C chemokine receptor type 5 (CCR5). CCR5 serves many functions in the human immune response and is a co-receptor utilized by HIV-1 for entry into immune cells. Therapeutics targeting CCR5 generally involve gene editing techniques including CRISPR, CCR5 blockade using antibodies or antagonists, or combinations of both. Here we review the efficacy of these approaches and discuss the potential of their use in the clinic as novel ART-independent therapies for HIV-1 infection.

## 1 Introduction

Human immunodeficiency virus type 1 (HIV-1) infection has been a global health problem for over 30 years, affecting more than 37 million people worldwide today (1). The search for a cure is challenged by the inter- and intra-patient diversity of HIV-1 as well as the establishment of latently infected cellular reservoirs that can remain latent for many years (2–8). The advent of antiretroviral therapy (ART), which consists of drugs that inhibit viral replication by targeting different HIV-1 proteins, has enabled control and prevention of newly infected cells. However, ART does not target latently infected cells since they are not actively transcribing HIV-1 genes nor does it resolve many of the immune dysfunctions caused by HIV-1 infection (7, 8). Cessation of ART thus leads to viral rebound or a return to uncontrolled viral replication in the HIV-1-infected individual, an outcome currently only avoided by life-long ART adherence. Due to this as well as the cost, side effects, and possibility of ART-resistant HIV-1 strains emerging, there is a need for novel therapeutics that can more efficiently allow long-term control of HIV-1 infection (7, 8). A therapeutic that can additionally prevent ongoing establishment of latent HIV-1 reservoirs would also make a cure more feasible.

A hope for an HIV-1 cure transpired with news of the Berlin patient, Timothy Ray Brown. Brown was an HIV-1-positive individual who received an allogeneic hematopoietic stem cell transplant as a treatment for relapsed leukemia. The transplant caused his HIV-1 viral load to decrease to undetectable limits (8, 9). The reason for this was found to be that the stem cell donor was homozygous for C-C chemokine receptor type 5 (CCR5) Δ32. This 32-base pair deletion in the CCR5 allele provides a mutation for the CCR5 gene, which encodes the CCR5 that is used as a co-receptor by HIV-1 for attachment and entry into the host cell (8, 9). More recently, another HIV-1 individual, Adam Castillejo, underwent a similar but less toxic version of allogeneic hematopoietic stem cell transplant from a homozygous CCR5Δ32 donor. Thirty months after analytical treatment interruption, the London patient, as he has been designated, has no detectable viral load in any of the examined regions including the peripheral blood, intestinal tissue, CSF, and lymph nodes. This led the authors to conclude that this patient represents a model for HIV-1 cure (10, 11). Targeting of the CCR5 receptor to render host cells less susceptible to infection or possibly resistant to infection may allow for more efficient inhibition of HIV-1 infection, in particular if combined with other anti-HIV-1 approaches.

The cases of the Berlin and London patients led many researchers to investigate other feasible methods for targeting CCR5 and their potential to serve as an HIV-1 cure for many other patients. Studies have investigated the inhibition of extracellular CCR5, through small molecule inhibitors or monoclonal antibodies, as well as the prevention of CCR5 expression, through gene editing techniques such as RNA interference, Transcription Activator-Like Effector Nucleases (TALENS), Zinc Finger Nucleases (ZFN), and Clustered Regularly Interspaced Short Palindromic Repeats (CRISPR). Recently, CRISPR has gained more interest as efficacy and lack of off-target effects (edits in other regions of the genome) allowed for a more convenient and sustained prevention of CCR5 expression, providing some benefits over other therapies targeting extracellular CCR5.

However, as a chemokine receptor with important roles in inflammatory signaling pathways, CCR5 is expressed on various immune cell types in addition to CD4+ T cells, the primary host cell targets of HIV-1 (8). While there remain challenges in determining long-term efficacy and safety of CCR5 targeting, investigational studies demonstrated some clinical success in suppressing HIV-1 infection. In this review, we highlight the biological functions of CCR5, summarize methods investigated for ablation of CCR5 in these studies, and evaluate the potential of their approaches to serve as a therapeutic for an HIV-1 cure.

## 2 Expression and Function of the CCR5 Receptor on White Blood Cells

### 2.1 Function and Prevalence on Immune Cell Populations

**Figure 1:**
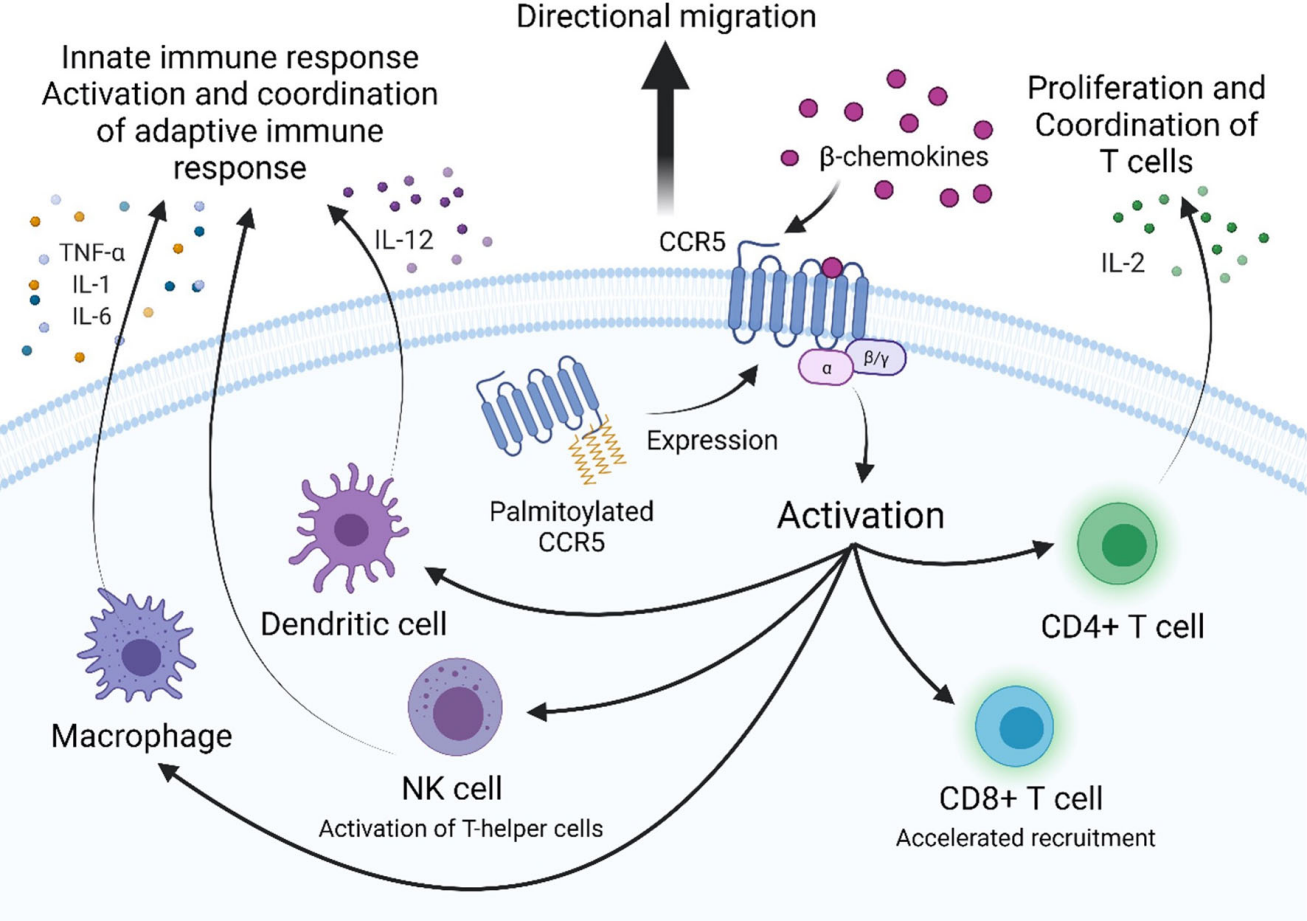
CCR5 is a G-protein coupled receptor that is involved in activation and coordination of the innate and adaptive immune response. Palmitoylation of multiple cysteine residues in the C-terminal domain target CCR5 to lipid rafts in the plasma membrane to participate in extracellular signaling. β-chemokines bind to extracellular domains of CCR5, activating it and inducing downstream signaling. CCR5 expression is required for directional migration and coordination of cells of the innate and adaptive immune response along a chemotactic gradient to sites of infection. CCR5-dependent secretion of pro-inflammatory cytokines by macrophages (TNF-α, IL-1, and IL-6) and dendritic cells (IL-12) activate the adaptive immune response. CCR5-dependent secretion of IL-2 by activated CD4+ T cells induces proliferation and activation of effector, memory and regulatory T cells. CCR5 is required for the accelerated recruitment of effector and memory CD8+ T cells to sites of infection.

CCR5 is an integral membrane protein expressed on various white blood cells (leukocytes) including cells of the monocytic lineage. When expressed on leukocytes, CCR5 serves as a receptor for inflammatory β-chemokines, which are produced by nearly every cell type during infection or injury and signal through G protein–coupled receptors (GPCRs). The chemokine ligands of CCR5 include Regulated on Activation, normal T-Expressed and Secreted (RANTES; CCL5), Macrophage-Inflammatory Protein-1α (MIP-1α; CCL3), and MIP-1β (CCL4). CCR5 is expressed on macrophages, Dendritic Cells (DCs), and Natural Killer (NK) cells, which are cells of the innate immune response, as well as on T and B cells of the adaptive immune response (12). Expression of CCR5 and chemokine binding exert downstream effects in a cell type-specific manner, which ultimately coordinate the migration of activated leukocytes, lead to secretion of pro-inflammatory cytokines, and stimulate cells of the innate and adaptive immune response (**Figure 1**).

Macrophages are a critical part of the innate immune response that recognize foreign pathogens and secrete antiviral cytokines and type I interferons (IFN-α and IFN-β), which inhibit viral replication by stimulating expression of interferon-stimulated genes (ISGs) that induce an antiviral state within the cell (13). A more immediate response is triggered by activation of CCR5, which was shown to induce expression of inflammatory genes iNOS, COX-2 and IL-1β through activation of nuclear factor kB (NF-κB) and secondary pathways *via* MAPKs ERK, JNK and p38 (13). Additionally, secretion of proinflammatory cytokines TNF-α, IL-1, and IL-6 can also occur. These trigger apoptosis, activation of NK cells, and activation and differentiation of T and B cells, respectively (13). Furthermore, β-chemokine binding to CCR5 is required for directed migration of macrophages (14, 15). This was demonstrated using a mouse model of hepatotoxicity, in which a CCR5 knockout decreased infiltration of macrophages to sites of damage, with production of TNF-α, and iNOs synthesis (16). Together, CCR5 expression on macrophages is an important component of the innate immune system for nitric oxide (NO) production, prostaglandins production, production of proinflammatory cytokines, and activation and coordination of both the innate and adaptive immune response.

For DCs, another cellular derivative of the monocytic lineage, CCR5 is involved in their cell migration to the lymph nodes and subsequent stimulation of naïve T cell differentiation into effector T cells in response to the chemokine CCL4 (17). Activated dendritic cells activate specialized T helper cells and NK cells and induce IFN-γ secretion by IL-12 synthesis and secretion in a CCR5-dependent manner (18, 19). Knockout of CCR5 and treatment with anti-CCL4 antibodies in mice was found to significantly reduce, but not completely abrogate, mobilization of DC precursors into the circulation in response to bacterial infection (20). Consequentially, the monocytic lineage plays a key role in the host defense against pathogens as well as immune regulation among other processes, which reflects the potentially integral function of CCR5 in these diverse processes (21).

NK cells, lymphocytes of the innate immune response, secrete IFN-γ to stimulate macrophages and increase expression of MHC II and chemokines to coordinate antigen-specific CD4+ and CD8+ T cells. NK cells also express CCR5 which is necessary for the control of NK cell trafficking in response to infection and coordination of the immune response (22). A study of influenza infection demonstrated that CCR5-deficient mice were more susceptible to infection and exhibited lower levels of NK cells trafficked to sites of viral infection (23). In the adaptive immune response, CD4+ and CD8+ T cells acquire CCR5 during the activation process. In peripheral blood, CCR5 is expressed on circulating memory CD4+ T cells, while in the thymus CCR5 is not expressed on CD3^-^ immature thymocytes (24–26). Similar to innate immune cells, chemokines coordinate T cell migration into lymph nodes and inflamed tissues. Activated CD4+ T cells orchestrate the immune response by secretion of IL-2, the T cell growth factor, which upregulates CD25 expression, a necessary step in activating and inducing proliferation of effector and memory T cells as well as regulatory T cells. This function is dependent on chemokine stimulated CCR5 intracellular Ca^2+^ transactivation of NFAT and subsequent IL-2 expression, which has been studied in CCR5-deficient mice, biologically relevant cell lines, and primary human T cells (27). Functional expression of CCR5 on antigen-specific memory and effector CD8+ T cells in response to β-chemokines has also been characterized. CCR5 is suppressed during differentiation of CD8+ thymocyte to naïve CD8+ T cells and to resting memory CD8+ T cells but expressed after differentiation to memory CD8+ T cells. CCR5-, but not CXCR3-deficient mice confirm that surface expression of CCR5 is required for the accelerated recruitment of CD8+ T cells to sites of respiratory viral infection to deliver cytotoxic IFN-γ (24, 28). In an LCMV infection of CCR5- and CXCR- deficient mice, CD8+ T cells were still able to infiltrate the CNS, but with a delay, and interestingly augmented generation of effector CD8+ T cells, supporting the thought that the cells can still migrate but not in an accelerated manner (29). The effector and memory CD8+ cells use CCR5 to follow a chemotactic gradient and exert their effect as well as contribute to controlled proliferation and activation. The diverse functions of CCR5 thus help mobilize and orchestrate the inflammatory response which is a necessary process that allows both the innate and adaptive immune system to protect the host against invading pathogens (**Figure 1**).

### 2.2 Structure and Transcriptional Regulation of Expression

**Figure 2:**
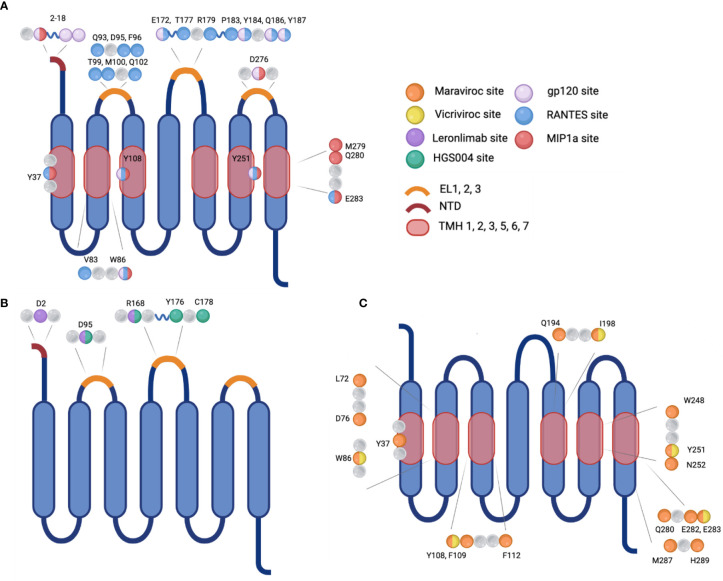
Visualization of the sites of interaction on CCR5 for natural ligands, HIV-1 gp120, monoclonal antibodies, or small molecule inhibitors. **(A)** Binding sites for the natural ligand RANTES or HIV-1 gp120. **(B)** Binding sites for monoclonal antibodies Leronlimab or HGS004, and **(C)** Binding sites for small molecule inhibitors Maraviroc or Vicriviroc. Mutation at selected amino acids inhibit interaction between binding molecule and receptor. EL, extracellular loop; NTD, N-terminal domain; TMH, transmembrane helical bundle.

Chemokine receptors are a family of seven transmembrane-spanning GPCRs of which the structure is conserved and characterized by a N-terminal extracellular region and C-terminal cytoplasmic region as well as seven α-helical hydrophobic membrane spanning domains, and three extracellular (ECL1-3) and intracellular (ICL1-3) loop segments (30). Several conserved amino acids in the extracellular regions compose the active site of CCR5, which is the site of ligand recognition and plays a major role for HIV-1 co-receptor function. They include a tyrosine rich motif in the N-terminal domain (NTD) and amino acids in the first and second ECLs of CCR5 (**Figure 2**) (30, 31). Sulfation of tyrosine residues in the NTD of CCR5 are required for binding ligands and the HIV-1 envelope protein gp120, which has been elucidated by NMR spectroscopy of this important CCR5 domain with RANTES/CCL5 (31, 32). Ligand binding is a two-step process. Site-directed mutagenesis and molecular docking have shown that core domains of CCL5 interact with ECL1, ECL2 and the NTD of CCR5 initially and the N-terminus of CCL5 interacts with the transmembrane helical (TMH) bundle of CCR5 (33). Two disulfide bridges linking together ECL1 and ECL2 (C101-C178) and ECL3 to the N-terminus (C20-C269) are required for maintaining the structural integrity necessary for the TMH bundle to associate closely together and form a binding pocket upon ligand binding, as determined by molecular modeling, ligand docking, and cryo-EM (33–35). CCR5 has been shown to be present in lipid rafts, a site for intracellular signaling; multiple palmitoylation of cysteine residues and a membrane-proximal basic amino-acid rich domain within the cytoplasmic tail facilitate downstream signaling, expression, and targeting to the cellular membrane (36, 37). The C-terminal domain (CTD) is also crucial for interaction with heterotrimeric G-proteins. Ligand binding induces conformational changes and desensitization by PKC/GRK dependent phosphorylation of the CTD and ICL3 followed by internalization of CCR5, and recycling to the surface after ligand removal. Additionally, the conformational changes induce secondary signaling pathways PI3K/Akt and MAPK/ERK *via* release of G-protein subunits and interaction with effector molecules to recruit cytotoxic lymphocytes and activation of antigen-specific T cells (38, 39).

CCR5 expression is activated by transcriptional regulators in response to cell stimulus. The gene encoding CCR5 has two functional promoter regions termed Pd (downstream) and Pu (upstream), named relative to the location of the transcription start site (40). Distributed among these sites are potential binding sites for several interferon stimulated response elements (ISREs), kB factors, and cAMP-response elements (CRE elements), which bind interferon regulatory elements (IRFs), NF-κB, and the common activator of transcription CREB-1 (CRE-binding protein), respectively (5, 41–47). These promoter elements were shown to bind to their activators *in vitro*, but ultimately the IRF and NF-κB sites were nonfunctional as determined by luciferase reporter assays after stimulation by IFN-γ and TNF-α and LPS, respectively (48–50).

### 2.3 Redundancy and Impact of Downregulation or Knockout

A 32-bp deletion in the CCR5 coding region (CCR5Δ32) has been reported to protect a homozygous individual from HIV-1 infection and delay disease progression in a heterozygous individual. CCR5Δ32 leads to a frameshift after amino acid 184 in ECL2, disrupting the open reading frame and affecting critical sites of post-translational modifications in the CTD. This results in a loss of critical serines and threonines, which are residues that normally become phosphorylated by kinases and participate in downstream signaling, and loss of cysteine residues, which normally become palmitoylated and are necessary for trafficking the receptor to the cell surface. Disruption of the CTD causes a sequestration of mutant CCR5 in the endoplasmic reticulum and Golgi, which prevents its expression at the plasma membrane on cells of a CCR5Δ32 homozygous individual (37, 51, 52).

Individuals who are homozygous for the CCR5Δ32 allele are reported to be resistant to HIV-1 infection, but these individuals only make up 1% of the human population (53). These individuals experience a slower loss of CD4+ T cells early in infection (54). There is an increased frequency of CCR5Δ32 heterozygotes among people living with HIV-1 who are classified as long-term non-progressors (LTNPs), also known as elite controllers. Transmission studies of CCR5Δ32 show that homozygous individuals have a high level of protection from HIV-1 infection, while those who are heterozygous have partial protection (55). The frequency of the CCR5Δ32 allele was assessed using samples from 87 countries and found to range from the highest allele frequencies (AFs) of 16.41%, 15.63% and 15.09% from Norway, Estonia and Latvia, respectively; while the lowest AFs were from Eritrea (0.26%) and Ethiopia (0%) (56).

The CCR5Δ32 allele has not been the only polymorphism of CCR5 described to influence susceptibility to HIV-1 infection. Polymorphisms in the regulatory, promoter, and coding regions of CCR5 influence transcription factor binding and levels of expression and have been shown to affect the risk of acquiring HIV-1 and the rate of disease progression to AIDS (57–59). These have been grouped into major human haplogroups (HH) based on the combination of cis-regulatory regions A29G, G208T, G303A, T627C, C630T, A676G, and C927T: A, B, C, D, E, F1, F2, G1, and G2 (57, 60). Haplogroup C (HHC) and haplogroup E (HHE) are the most frequent HHs in HIV-1-infected patients across many races and ethnic populations studied (61–63). Both HHG2, which includes the Δ32 allele (rs333), and HHF2, which includes CCR2 V64I (rs1799864), have been associated with resistance and slow progression to AIDS (57, 60, 64). HHE, which includes promoter variants rs2856758 (G29A) and rs1799987 (G303A) is associated with increased promoter activity as well as increased CCR5 expression, susceptibility to HIV-1 infection, and accelerated AIDS progression (57, 60, 63, 64). Indeed, among a cohort of children the 303A/A genotype was correlated with increased rates of disease progression. HHE was also underrepresented in elite controllers as compared to progressors from a black South African ART-naïve HIV-1-infected cohort (58, 65). The HHE 29G and 303G polymorphisms have been linked to decreased surface expression and reduced *in vitro* infectability, determined by flow cytometry of CD4+ T cells and monocytes of exposed seronegative high-risk individuals, though this may be linked to ethnic background as results were not significant for non-Caucasian individuals (66, 67). Among a South American cohort of HIV-1-exposed seronegative (HESN) individuals in serodiscordant relationships, who despite repeated exposure to HIV-1 remain seronegative, CCR5Δ32 was not the protective factor and was found in similar frequencies among HESNs, seropositive individuals and healthy controls of this cohort (61). However, frequencies of SNPs in the promoter, such as A29G was significantly different between controls and seropositive individuals, as well as frequencies of CCR5 haplogroups, HHF1 was found only among healthy controls and HHF2 had a higher frequency among controls compared with seropositive individuals (61). Thus, variants in the promoter of CCR5 have been shown to affect transcript levels and cell surface expression of CCR5 and therefore susceptibility to HIV-1 infection.

These studies on the impact of downregulated or diminished expression of CCR5 in individuals as well as cases such as the Berlin patient, who was infected with HIV-1 and received a hematopoietic stem cell transplant from a CCR5Δ32 homozygote, have suggested the possibility of engineering an HIV-resistant immune system through the suppression of CCR5. However, the impact of CCR5 inhibition on the orchestration of the immune response first needed to be carefully considered before this approach can be deemed feasible. Despite the many diverse functions of CCR5 in the immune response, analyses of whole-genome genotyping and whole exome sequencing data from the UK Biobank and US patient cohorts show that there is no evidence of correlation between mortality and CCR5Δ32 homozygosity (68, 69). These studies were conducted in response to a previous and now retracted study that showed the opposite (70). This may be explained by redundancy in chemokine receptor function. Studies investigating the effect of inhibiting CCR5 expression, through a knockout, elucidated that other receptors may substitute for CCR5 functions. β-chemokines CCL5 and CCL3 can bind to other receptors in the chemokine receptor family such as CCR1 and CCR3. CCL4 can bind CCR8, but CCR1 and CCR3 are not present on T cells and CCR8 is not present on macrophages (71–73). CD8+ T cells can preserve their functional recruitment to sites of infection without CCR5 through expression of CXCR3 although this is delayed compared to when CCR5 is present (29). Additionally, after CCR5 knockout in mice and induction of hepatotoxicity, macrophages successfully migrated to the liver and those of knockout mice were significantly increased for expression of CCR2 preserving chemokine chemotaxis (16). Infected mice lacking CCR5 exhibited increased and accelerated CD4+ T cell proliferation augmenting disease progression, suggesting that loss of CCR5 negates a protective role of CCR5-mediated CD4+ T cell activation but is also not necessary for recruitment of immune cells (74). In this regard, CCR5 appears to play a complimentary rather than integral role in the immune response and its absence does not compromise the antiviral response due to the redundancy of chemokine receptors and their ligands.

## 3 Role of CCR5 Expression in HIV-1 Infection

### 3.1 Requirement of CCR5 for HIV-1 Entry Into Some Immune Cells

Viral envelope glycoproteins on the surface of the HIV-1 virion utilize the primary receptor CD4 and co-receptors from the chemokine receptor family, CCR5 or CXCR4, to gain entry into target host cells. The envelope glycoproteins are encoded by Env, and associate as trimers at the lipid membrane of the virion as non-covalently bound surface gp120 (SU) and transmembrane gp41 (TM) subunits.

In the first step of viral entry, the gp120 subunit binds to one or more CD4 primary receptors, triggering conformational changes in gp41 and exposing a chemokine receptor binding site which was previously occluded. The V3 loop gp120 residues interact with the residues within the chemokine binding pocket and in ECL1 and ECL2 of the co-receptor, CCR5 or CXCR4, and interacts with the N terminus which also contacts the bridging sheet of gp120 (**Figure 2**) (75–79). Sequential binding to CD4 and a co-receptor bring gp41 and gp120 closer to the target membrane triggering the domains of gp41 to undergo a complex folding to form a fusion intermediate involving a six-helix bundle. This allows gp41 to insert its highly hydrophobic fusion peptide into the lipid bilayer of the target cell membrane with the subsequent fusion of the two membranes and formation of a pore through which the viral capsid can enter into the cytoplasm of the infected cell (80).

HIV-1 gene expression is dependent on host transcription factors, such as NF-κB, Sp, CEBP, CREB, among many other cellular transcription factors (42–44, 47, 49, 81–83). In particular, NF-κB is activated in response to T cell activation upon antigen recognition and leads to enhanced HIV-1 replication and cellular differentiation to effector T cells which release into peripheral blood, a process also known as thymopoeisis. Activated CD4+ T cells are the main cell type that support HIV-1 infection. Direct infection of naïve T cells is less efficient, in part, due to undetectable levels of CCR5 expression (84). A subset of activated cells differentiates to resting memory T cells and some eventually alter their pattern of gene expression and revert to resting memory T cells to enable long-term survival and induce a rapid response after re-exposure to antigen. HIV-1 stably integrated into the host genome of memory T cells or those that have circumvented the fates of activated T cells and reverted to memory T cells, are affected by the lack of transcription and do not express viral RNA, this is termed post-integration latency (85, 86). Pre-integration latency can also occur when HIV-1 infects naïve T cells which are quiescent, blocking the reverse transcription and integration of HIV-1 into the host genome, and later transition to effector or memory cells (87). In either case, HIV-1 transcription and translation can be rescued by activation of naïve cells leading to infected effector and memory cells, or by re-activation of memory cells. Thus, HIV-1 latency and a latent reservoir consist mainly of CCR5-expressing cells and can occur due to (i) infection of activated memory T cells that persist in a memory T cell state, (ii) infection of resting memory T cells, (iii) infection of an activated thymocyte in the transition to naïve T cells, or (iv) infection of activated T cells that transition back to resting memory T cells (5, 14, 85, 88).

### 3.2 CCR5 Versus CXCR4 Co-Receptor Use Among Variants and Relation to Disease Stage

HIV-1 tropism is classified by the co-receptor used by the variant; R5 viral strains utilize the CCR5 CC-chemokine co-receptor, X4 strains utilize the CXCR4 CXC-chemokine co-receptor, and dual-tropic R5X4 variants have the ability to use both co-receptors though with a greater affinity for CCR5 or CXCR4. Strains that are exclusively R5 predominantly infect monocyte-derived macrophages and memory CD4 cells, which are the prime targets of HIV-1 early in infection, while exclusively X4 strains predominate at a later stage and prefer naïve and resting T cells (44, 84, 89). Early infection is predominantly achieved by R5 tropic viruses because of the relatively high surface expression of CCR5 than CXCR4 on CD4+ memory T cells and immature dendritic cells which determines the efficiency of viral entry, as well as a higher affinity for CD4 (84, 90). HIV-1 transmission by R5 strains is more efficient than X4 strains, as is viral replication. This is supported by studies of people living with HIV-1 (PWH) who are not on antiretrovirals being infected mainly by R5 strains (80-91%), with some dual-tropic (9-20%) and very rarely X4 strains (>1%) (91, 92). In contrast, among PWH on antiretroviral therapy (ART), which clear the pool of infected CD4+ cells, R5/X4 and X4 strains are more common. In approximately 50% of HIV-1 infections, a co-receptor switch by mutation at the site of interaction in variable loops of gp120, especially V3, leading to alteration of N-linked glycosylation sites enables the switching of R5 to X4 tropism (75, 93, 94). X4-utilizing viruses are associated with a more rapid decrease in CD4+ cell count and an accelerated rate of disease progression and mortality in contrast to R5 tropic viruses (91, 95). However, immune activation and progression are not a result of the switch to X4 tropism but rather are a consequence of CD4+ T cell activation depleting host target cell availability, driving the target to naïve T cells allowing X4 strains to predominate later in the course of infection (96, 97). Long-term non-progressors are a group of PWH able to maintain stable CD4 cell counts and remain asymptomatic without ART. They exhibit lower amounts of CCR5 expression on memory CD4+ T cells compared to normal progressors and healthy controls while CXCR4 expression was similar compared to normal progressors but significantly higher than healthy controls (98, 99). CCR5 and high levels of CCR5 are associated with acute and early HIV-1 infection and rapid disease progression, while low CCR5 expression protects from virus infection (100).

## 4 Mechanisms of Targeting CCR5 to Inhibit HIV-1 Disease Progression

### 4.1 Extracellular CCR5 Blocking Methods

#### 4.1.1 Small Molecule Inhibitors

Given that CCR5 can be utilized by HIV-1 to enter and infect immune cells, extracellular methods of inhibiting the interaction of gp120 with CCR5 have been developed (**Table 1**). Targeting and preventing this interaction has been mainly done with the use of small molecule inhibitors, which generally work by inducing conformational changes to CCR5 thereby preventing fusion of the HIV-1 envelope with the cellular membrane (108). In contrast to many therapeutics targeting viral proteins, these inhibitors target the various components of the transmembrane CCR5 receptor protein on host cells.

**Table 1:** Overview of clinical trial outcomes of selected CCR5 antagonists in HIV-1 infection.

Study	N=	Intervention	Duration or Dose	Outcomes	Notes
Three-Year Safety and Efficacy of Vicriviroc, a CCR5 Antagonist, in HIV-1-Infected, Treatment-Experienced Patients (NCT00082498)	118	Failing Background Therapy + Vicriviroc	5, 10, 15 mg/day up to 3 years	1) 46% were suppressed <50 copies/mL after 24 weeks 2) Through the third year 49% did not rebound	• 11% developed malignancies • 29% of patients had mixed tropism • 5.1% developed resistance (101)
Vicriviroc in combination therapy with an optimized regimen for treatment-experienced subjects: 48-week results of the VICTOR-E1 phase 2 trial (NCT00243230)	114	Ritonavir + Vicriviroc or Placebo	20 or 30 mg/day for 48 weeks	1) Mean viral load change for intervention groups was 1.75, 1.77 log_10_ copies/mL compared to placebo 0.79 log_10_ copies/mL 2) Mean CD4 count increased 102, 136 in treated groups and 63 in placebo	• Four subjects discontinued due to adverse events • Mild elevations in liver tests were observed (102)
Clinical Trial Vicriviroc in HIV-Treatment Experienced Subjects (NCT00523211)	506	Background Therapy + Vicriviroc	30 mg/day for 48 weeks	1) Dual therapy with Vicriviroc achieved suppression more frequently than dual therapy without Vicriviroc 2) At 48 weeks no additional efficacy was seen in patients receiving 3+ drugs	• 60% of patients were on 3 or more antivirals • Adding Vicriviroc did not provide additional efficacy gains • Included only patients with CCR5-tropic infections (103)
Maraviroc as an Immunomodulatory Drug for Antiretroviral-treated HIV Infected Patients Exhibiting Immunologic Failure, Phase 4 (NCT00735072)	45	Maraviroc + Efavirenz or Tipranavir	150, 300, 600 mg twice/day 48 weeks	1) Maraviroc group experienced less of a decline in CD4+ T cell count and an increase in circulating CD8+ cells 2) Low-level viremia decreased on average 48% and 52% in placebo and intervention	• Maraviroc treatment appeared to induce re-localization of activated CD8+ cells from the gut to the periphery (104)
Maraviroc as intensification strategy in HIV-1 positive patients with deficient immunological response (NCT00884858)	100	HAART + Maraviroc	Scaled doses 150-600 mg twice daily up to 48 weeks	1) Maraviroc did not display an advantage in improving CD4+ counts 2) CD8+ counts improved in maraviroc intensified groups	• Study focused on patients with decreasing CD4 counts (105)
Study of PRO 140 by Subcutaneous Administration in Adult Subjects With HIV -1 Infection (NCT00642707)	44	Subcutaneous Leronlimab	62 mg or 324 mg/week for 3 weeks or 324 mg biweekly	1) Log_10_ reduction of 0.23, 1.37 and 1.65 accordingly	• Doses were well tolerated • Serum concentrations were stable through day 8 (106)
A Phase 2a, Randomized, Double-Blind, Placebo-Controlled Study of PRO 140 by Intravenous Administration in Adult Subjects With HIV-1 Infection (NCT00613379)	31	Intravenous Leronlimab	Single 5 or 10 mg/kg infusions	1) Average maximum reduction in viral load was 1.8 log_10_ 2) Receptor occupancy remained above 85% in both groups day 3 through day 29 but change in occupancy was not significant by day 59	• Patients had been off ART for 3 months or more, had viral loads >5000 copies/mL and CD4 counts >300 (107)

These trials reflected common use of the intervention in clinical practice.

In the early 2000s, several drugs were designed as orally available small molecule CCR5 inhibitors but did not complete stage 3 clinical trials. One of the earliest small molecules, Aplaviroc, was discontinued due to evidence of hepatoxicity in four patients (108). Another CCR5 antagonist, Vicriviroc, showed efficacy in reducing viral loads of treated patients by about one log over 24 weeks, but *in vivo* resistance developed in one patient (**Table 1**). It has not been approved for clinical use because of a potential link to the induction of hematological malignancies in five patients (101).

Later in 2007, the drug candidate Maraviroc was approved for clinical use to act as a non-competitive inhibitor of the CCR5 receptor (**Table 1**). It is the only CCR5-blocking drug approved for clinical treatment of HIV-1 infection (109). The transmembrane hydrophobic binding site for Maraviroc is not the same used by the major chemokines or gp120. Maraviroc stabilizes a conformation of the CCR5 receptor that is unable to be bound by gp120 (109). HIV-1 is still able to interact with the receptors allosterically bound by Maraviroc but not use them efficiently, leading to suppression of infection. Clinical trials have shown Maraviroc can reduce viral load in treatment naïve patients and patients previously teated with ARRT who are positive for only CCR5-utilizing HIV-1 strains (101). Despite promising clinical trial results, mutations in the highly variable V2 and V3 loop region of viral gp120 have been reported, which result in a recovered CCR5 receptor usage even with the presence of Maraviroc at the binding site (101). Finally, potential changes in viral tropism to utilize CXCR4 as a co-receptor have been of concern, but diagnostic limitations make it difficult to discern novel Maraviroc resistance within the host from the emergence of a pre-existing CXCR4-tropic strain (108, 109). For these reasons, current clinical use trends towards a treatment experienced cohort where ART strategies have failed. In many infected patients, Maraviroc has been added to their regimens as a treatment intensification approach due to low CD4 counts (110). Of note, in a study assessing efficacy and safety of Maraviroc showed slightly increased CD4 counts through 9 months of treatment and appeared to increase naïve CD8+ T cells in the digestive tract, highlighting the potential benefit of restoring immune function by targeting infection-associated inflammation in lymphoid tissues (111).

Other more recent small molecule inhibitors in development include GRL-117C, which demonstrated inhibition of R5-utilizing HIV-1 (108). Interestingly, this study also implicated CCR5 inhibitors in additional benefits for treatment of HIV-1 infection including immunomodulation and even latency reversal. Overall, while small molecule inhibitors confer some protection against HIV-1 infection, results of their treatment usage demonstrate a more feasible therapy is needed that would limit onset of resistant HIV-1 strains as well as be formulated in a way that patients can take easily.

#### 4.1.2 Cases of Natural Antibodies to CCR5

Individuals exposed but uninfected and well-suppressed infected individuals have been shown to have detectable CCR5 antibody. These antibodies have been found in circulation and in mucosal surfaces, a key site for HIV-1 transmission (112). The natural antibodies inhibit HIV-1 infection via binding to the extracellular loop 1 (EL1) of CCR5, inducing receptor internalization (110). Interestingly, CCR5 antibodies were also found in almost a quarter of long-term non progressors, and *in vitro* analysis showed CD4+ T cells from these patients were not susceptible to CCR-5 tropic viruses. Studies have observed no deleterious immune impact in individuals seropositive for anti-CCR5 and these proteins may confer enhanced viremic control *in vivo* (112–114).

#### 4.1.3 Development of Monoclonal Antibodies

In addition to small molecule inhibitors, monoclonal antibodies targeting the CCR5 receptor are being developed and investigated for use in treatment of HIV-1 infection and pre-exposure prophylaxis (PrEP). These antibodies are intended to bind the CCR5 receptor to inhibit gp120 interacting with the co-receptor (**Figure 2**). The drug Leronlimab is a humanized anti-CCR5 IgG4 monoclonal antibody that is delivered subcutaneously or intravenously (**Table 1**) (115). Preliminary studies have shown that contrary to natural antibodies, Leronlimab is able to bind the N-terminal domain of EL2 on the CCR5 receptor, the same binding site used by gp120 (**Figure 2**). This loop is thought to be a well conserved area of CCR5 encoding genes (108). Another CCR5 antibody HGS004 directed at the same area of CCR5 has also show *in vitro* and *in vivo* efficacy in infected patients. However, a linear dose-dependent response was not observed and only about 50% of patients showed a viral load decrease of greater than one log two weeks after a single dose (116).

Studies in rhesus macaques showed dose-dependent protection from CCR5-utilizing infection following injections of Leronlimab subcutaneously. Additionally, 50 mg/kg prevented HIV-1 infection in all sites for all subjects, while just 10 mg/kg prevented infection in rectal tissue in all but one subject (117). In Phase 2 clinical trials in individuals with solely CCR5-utilizing HIV-1 intravenous Leronlimab infusion was well tolerated. Dosage as low as 5 mg/kg elicited maximum antiviral effects around 14 days post injection with greater than 1.8 log viral load reduction (118). In this same study, viral load rebounded to near baseline in all dosages around day 40 post-injection, highlighting a need for sustained treatment. No evidence of resistance or switched tropism was observed while only mild side effects were encountered with this medication, and it has been given a fast track status by the US Food and Drug Administration (FDA) (119).

Of the monoclonal antibodies directed at CCR5 that have been investigated, Leronlimab has achieved the most sustained receptor occupancy. Promising infection prevention and antiviral data has been gathered from clinical trial and macaque studies. Patients exhibited 85% receptor occupancy through day 29 post-infusion of both 5 and 10 mg/kg doses (106). Additionally, while Leronlimab could benefit other neurological diseases, the issue of the viral reservoir will likely not be well addressed by these monoclonal antibody treatments. Studies report 70-75% receptor occupancy in Leronlimab-treated macaques (120). Long-term treatment sustainability and standardized treatment protocols have yet to be determined, though several patients have seen continuous suppression for over two years.

### 4.2 Alteration of CCR5 Expression as a HIV Therapeutic

#### 4.2.1 RNA Interference

The original concept of RNA interference as a gene editing tool was noticed in *C. elegans* and now includes three distinct tools: short hairpin RNA (shRNAs), short interfering RNA (siRNA), and microRNA (miRNA). While similar in that they each modulate the expression of a gene target, they each have some relevant differences. shRNAs are similar to siRNAs in that they target only one mRNA transcript, but different in that the shRNA coding sequence is stably integrated into a cell’s genome allowing for long-term expression. In contrast, siRNAs are only expressed in the cytosol which is conducive for transient knockdown of the designated mRNA. miRNAs, while initially only found endogenously in cells, have recently become synthesized artificially (121). miRNAs are distinct in their structure, which does not fully compliment the target mRNA, allowing for multiple targets. All three of these have been evaluated in the knockdown of CCR5 for therapy of HIV-1 infection.

Two main cell types have been primarily used in CCR5 knockdown experiments: hematopoietic stem and progenitor cells (HSPCs) and CD4+ T cells. HSPCs provide the advantage of differentiating into macrophages and CD4+ T cells that could be resistant to CCR5-utilizng HIV-1, which contributed to the success of therapy in the Berlin and London patients. However, practical usage of HSPCs in HIV-1-infected individuals is complicated by (i) the damage caused to HSPCs and hematopoietic function in bone marrow from their infection by HIV-1 (122), (ii) the damage to the differentiation potential of HSPCs caused by alteration of these cells (123), and (iii) the rarity of HSPCs and associated difficulty of culturing them *in vitro* (124).

Experiments done in HSPCs have shown significantly better results *in vitro*, compared to *in vivo*. Due to their non-dividing nature, research has focused on de-differentiating hematopoietic stem cells into induced-pluripotent stem cells (iPSCs) to provide a replenishing source of cells. One such study knocked down CCR5 in iPS- derived hematopoietic stem cells using a shRNA, these modified iPSCs then underwent directed differentiation back into hematopoietic stem cells and then end-stage macrophages, while CD4+ T cells were not generated. Above 99% iPSCs were observed to possess shRNA against CCR5, resulting in only 6.7% of macrophages positive for the receptor. These macrophages inhibited HIV-1 infection by more than 2 logs, compared to controls (125). However, CCR5 knockdown and engraftment of edited cells into mice is challenging, while no research to date has been published concerning shRNAs editing HSPCs which are then engrafted into mice. However, miRNAs have been used for this purpose. Myburgh et al. demonstrated more than 70% miRNA transduction into HSPCs using a lentiviral vector. This study’s promising results showed 11 of the 15 mice had hCD45+ cell engraftment above 5%, but all 11 of these mice displayed successful CCR5 knockdown below 20% of the control level in CD4+ T cells (126). However, there was evidence of viral escape of the YU-2 CCR5-utilizing infectious molecular clone in one mouse, leading to CXCR4-utilizing virus (126).

In contrast, peripheral CD4+ T cells are significantly more available for experimentation and prolonged proliferation *in vitro*. Additionally, some CD4+ T cell subsets have self-renewal properties similar to stem cells. Stem cell memory T cells, central memory T cells, and effector memory T cells are all capable self-renewal, thus sustaining any modifications made to them. Artificial miRNAs used in primary CD4+ T cells achieved a 39% successful CCR5 knockdown, and a near full reduction in viral load for the length of the eight day experiment (121). While CD4+ T cells and hematopoietic stem cells are the dominant models for gene editing, macrophages are also an important target for HIV-1 infection. In fact, CCR5-utilization is more relevant for macrophages which express high levels of CCR5, as compared to CD4+ T cells, but macrophages express low levels of CD4 and so are more relevant later on in infection when CD4+ T cells are depleted. However, the long-term viability of macrophage transduction with gene editing tools have been challenging, as macrophages are a mostly non-dividing cell type and they phagocytose viral vectors. As a result, there is significantly less research on cells of monocyte-macrophage origin. One effective approach to studying macrophages has been the use of HSPCs or iPSCs that are edited and then differentiated into macrophages. This approach provides a renewable source of CCR5-edited macrophages. Using this strategy, shRNAs were able to achieve above 99% CCR5 knockdown in iPSCs and maintained their modifications past the differentiation into end-stage macrophages (127).

**Figure 3:**
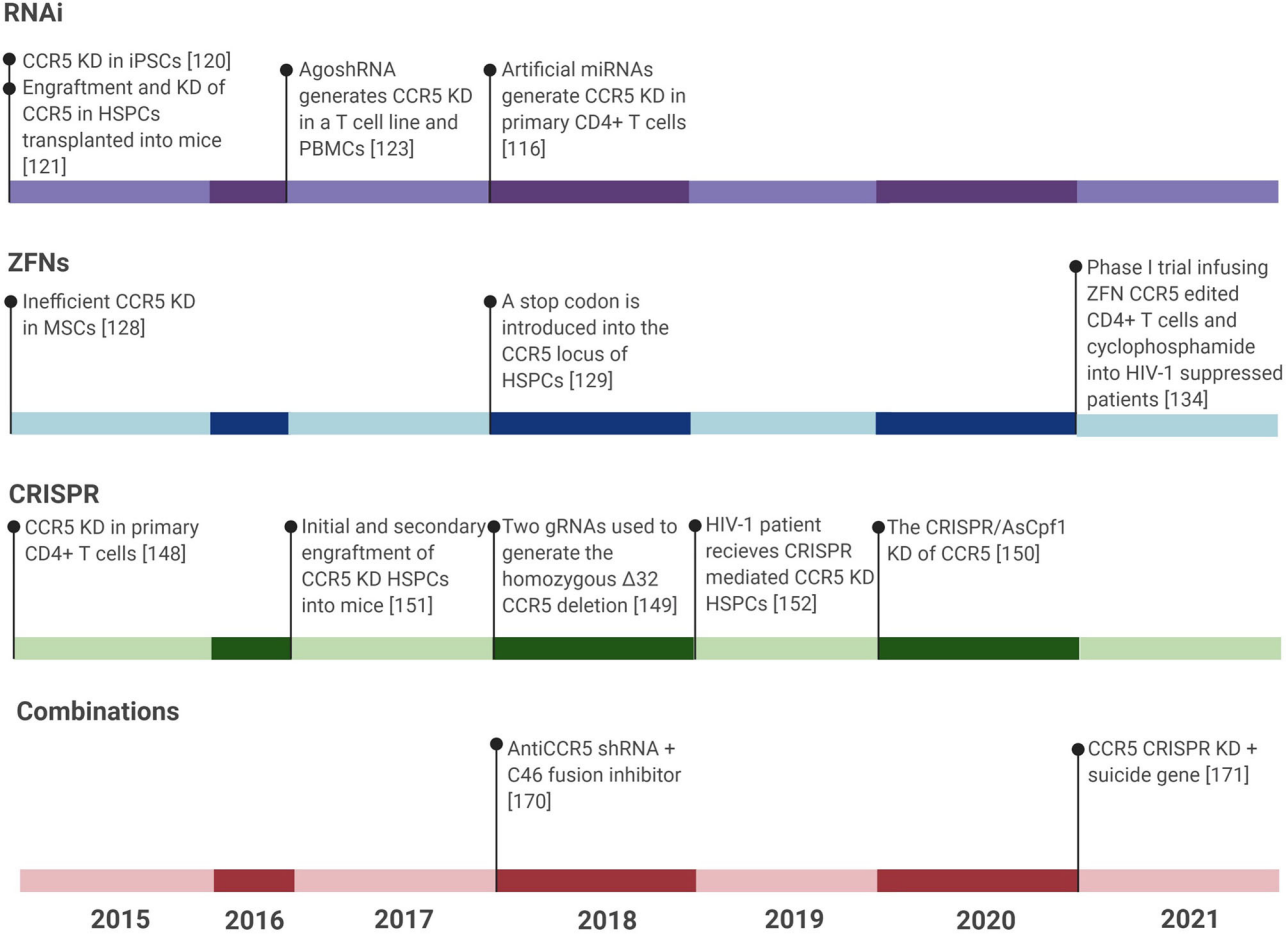
Timeline showing all CCR5 gene editing studies for treatment of HIV-1 infection in the last six years. Gene editing studies include RNAi tools, ZFNs, CRISPR, and combinations of treatments against HIV-1. TALENs were not included due to the publication of only 2 major studies on CCR5 editing using TALENs within the last six years. The last six years have seen the most published research in these areas and so any research prior to this has been left out of this figure. KD, Knockdown.

AgoshRNA is a relatively new type of shRNA that is smaller than typical shRNAs and is able to be expressed in monocytes unlike their predecessors. Their smaller size precludes agoshRNAs from being processed by the conventional Dicer, but instead leads to processing by Ago2. Monocytes lack Dicer, thus expanding the cell types available for expression. Anti-CCR5 AgoshRNAs have reduced the number of CCR5-positive cells to less than 20% in the PM1 T cell line and less than 40% in PBMCs. This reduction in CCR5 expression translated to no detectable replication of HIV-1 for the duration of a 25 day experiment, as measured in the PM1 T cell line. In addition, cytotoxicity was found to be negligible (128) (**Figure 3**).

Although a large amount of research exists with respect to the usage of RNAi tools they have largely fallen out of in favor due to their tendency to trigger an innate immune response, their transient nature, minimal ability to penetrate a cell, incomplete knockdown of the genes of interest, and frequent off-target effects. These disadvantages make development of a highly efficient, long-term therapeutic for HIV-1 infection that is based on inhibition of CCR5 expression through RNAi very unlikely, while more robust CCR5 targeting approaches can allow for better therapeutic outcomes.

#### 4.2.2 Zinc Finger Nucleases

In 1985, the zinc finger (ZF) was first identified as possessing an adaptable DNA recognition domain, Cys2His2-ZF, which showed promising DNA-binding results as a gene expression regulator. Cys2His2-ZF is the most common type of DNA-binding motif in eukaryotic transcription factors and constitutes 3% of the genes of the human genome due to its adaptable nature (129, 130). The modular design of ZFs permits numerous combinatorial possibilities for recognizing specific DNA and RNA sequences. ZFs were shown to have applications in biotechnology in 1994, when Choo et al. demonstrated a three-finger protein capable of blocking the expression of a human oncogene (131). Each zinc-finger unit selectively recognizes three base pairs (bp) of DNA and produces base-specific contacts through the interaction of its α-helix residues with the major groove of DNA. The zinc finger peptides are linked to the non-specific catalytic domain of the Fok1 endonuclease creating ZF nucleases (ZFN). Cleavage by Fok-I generates two 5′-overhang DNA ends. Because each zinc-finger unit recognizes three nucleotides, three to six zinc-finger units are assembled to generate a specific DNA-binding domain that recognizes a 6- to 18-bp DNA sequence. The target sequence specificity and recognition of ZFNs are influenced by three central aspects: (i) the amino acid sequence of each finger, (ii) the number of fingers, and (iii) the interaction of the nuclease domain. Both the DNA-binding and catalytic domains of ZFNs can be individually adjusted due to the flexible structure of ZFNs, thus facilitating the development of new ZFN designs with the necessary affinity and specificity for selected gene therapy applications (132).

As one of the first gene editing tools, ZFNs have had more time to be developed for clinical use. Taking a somewhat different approach, Manotham et al. demonstrated ZFN-mediated homology directed repair in bone marrow-derived mesenchymal stem cells (MSC). The advantage of MSCs is that they are relatively easily procured through bone marrow aspiration. Further, MSCs have one of the highest proliferation rates of any primary cell culture, contrary to CD4+ T cells and especially HSPC which are difficult by comparison to culture *in vitro*. In theory, editing MSCs using ZFNs should be an effective means to inhibit HIV-1 infection, but in practice editing efficiency is well below optimal. Manotham et al. found that, out of 10,236 cells that they had attempted to edit, only 6 cells were capable of proliferation and contained one allele of the CCR5 gene insertion (133). More recently, the same group attempted to introduce a stop codon into the CCR5 locus of HSPC cells using ZFNs. PCR indicated that only 0.5% of HSPCs contained the stop codon insertions within the CCR5 loci (134).

The translation of this type of research *in vivo* has yielded relatively positive results, with the promise of more to come in clinical trials. Holt et al. demonstrated that 11% of HSPCs engrafted into a mouse model contained the CCR5 disruption, which is a much higher frequency of edited cells found to engraft into mice compared to the insertion experiments by Manotham et al. which were not engrafted into mice, demonstrating how much more efficient simple cleavage is compared to introduction of a gene. This engineered protection led to undetectable HIV-1 RNA in the small and large intestine of mice at 12 weeks post CCR5-utilizing HIV-1_BAL_ challenge as measured by quantitative PCR (135). These studies have led to translation of this research into clinical trials, for example, the phase I clinical trial run at the City of Hope Medical Center is administering ZFN CCR5 modified autologous SB-728mR-HSPC to HIV-1 CCR5-utilizing infected patients to assess their safety and feasibility at inhibiting infection with CCR5-utilizing HIV-1. This study will conclude in 2022.

Although HSPCs have more long-term potential, the convenience of working with CD4+ T cells has made their use more widespread when using ZFNs. Mice transplanted with CCR5-negative CD4+ T cells from ZFN modification, showed reduction in HIV-1 replication. Perez et al. established that ZFNs effectively disrupt CCR5 in human CD4+ T cells and that this disruption provides sustained inhibition of HIV-1 infection *in vitro* and *in vivo* using the immunodeficient NOD/Shi-*scid/*IL-2Rγ^null^ (NOG) mouse model. Between 50-80% of CCR5 was observed to be mutated in GHOST-CCR5 cells as measured by the Surveyor assay. In primary cells, CCR5 disruption reached 40-60%. When human CD4+ T cells were infused into ten HIV-infected NOG mice, more than 50% of CD4+ T cells in eight of the ten mice contained the CCR5 disruption 50 days after engraftment. This led to a mean viral load of 8,300 copies/ml compared to 60,100 copies/ml in the control (136). Yi et al. expanded on this work in their study using resting T cells in which CCR5 expression was disrupted using ZFN, which were then transplanted into mice (137). The transplantation resulted in a 71% disruption frequency in the CCR5 of these cells in the mice. This modification of CD4+ T cells allowed inhibition of HIV-1 infection and resulted in a significant reduction in viral load, as measured by p24 ELISA and qRT-PCR. Furthermore, this also led to less reduction in CD4+ T cell counts (137).

These successful *in vitro* and *in vivo* studies led to clinical trials, where ZFN CCR5 modified CD4+ T cells were transplanted into HIV-1-infected patients that had predominant CCR5-utilizing virus and initially 13.9% of peripheral CD4+ T cells contained the CCR5 knockdown but at 42 months the concentration of peripheral CD4+ T cells with this modification had reduced to 1.7%. During a 12 week analytical treatment interruption (ATI) that began four weeks post-modified CD4+ T cell infusion, 4 patients had an average of 1.2 log_10_ decrease in viral load. However, by the end of the ATI, the median circulating CD4+ T cells had declined from 1849 per cubic millimeter at the start of the ATI to 872 per cubic millimeter (138). This same group recently published another phase I trial also using ZFN CCR5-edited CD4+ T cells and cyclophosphamide to increase the engraftment of the modified cells by depleting the presence of immune cells. Infusion of these cells was generally safe and well tolerated with no serious adverse effects throughout the 48 week experiment. Pretreatment with cyclophosphamide had no discernible influence on the time it took to virologic rebound, but a slight trend was observed in improved engraftment in those exposed to cyclophosphamide. One week post-infusion, the median frequency of ZFN CCR5-edited CD4+ T cells was 17%. Similar to previous studies, the overall trend of modified CD4+ T cells was that modified lymphocytes increased then decreased in the peripheral blood, likely due to cell localization to certain tissues or cell death. Notably, no significant increase or decrease in the viral reservoir was detected by intact proviral DNA assay (IPDA) following ATI. In summation, this study demonstrated that CCR5 knockdown CD4+ T cell infusions are safe and may delay viral rebound, but do not have any long-term effects on HIV-1 reservoirs (139) (**Figure 3**).

#### 4.2.3 TALENs

Transcription activator-like effector nucleases (TALENs) have structural similarities to ZFNs as they are heterodimeric nucleases that consist of a fusion between the Fok-I catalytic domain and a transcription activator-like effector (TALE) DNA-binding domain. The DNA- binding domain consists of an array of almost identical repeats of 33–35 amino acids. Each of these repeats independently recognizes one nucleotide through two amino acids called repeat variable diresidues (RVDs), and the recognition specificity is determined by the RVD. TALE modules differ from ZFs in that individual TALE modules seem to recognize DNA mostly independent of their adjacent modules (140, 141). A disadvantage to using TALENs is that the genes encoding the system are approximately three times the size of ZFNs, due to TALE motifs having a comparable size to ZFNs, but TALE motifs only recognize a single base, whereas ZFs recognize three to four bases. Additionally, the consistently repetitive sequences of TALE modules, with the exception of the RVDs, create difficulties in assembling the genes encoding TALENs in *E. coli* for replication. For the same reason, delivery of TALENs into mammalian cells using viral vectors is also difficult (142). Although TALENs were first described in 2010, before TALENs became a sustainable alternative to ZFNs, the CRISPR/Cas9 system was beginning to gain attention.

TALENs have sustained little interest in the gene editing field due to the complexity and expense, especially when compared to cheaper, simpler alternatives such as the many variants of the CRISPR system (143, 144). And for these reasons TALENs have rarely been used to target CCR5. What little research that has been done *in vitro* on CD4+ T cells demonstrates some off target effects leading to low levels of cytotoxicity, as well as high nuclease activity and specificity. No research using TALENs to disrupt CCR5 in HSPC has yet to be published as of this writing. The main TALEN tool developed, CCR5-Uco-hetTALEN, includes a heterodimeric Fok1-cleavage domain and almost completely reduces off-target effects, with the notable exception of the highly homologous CCR2 (145). This technology has advanced so much that it is now automated and can reliably generate the CCR5 knockdown in frequencies above 60% within primary T cells, 40% of which can be biallelic CCR5 mutations (146).

#### 4.2.4 CRISPR

The clustered regularly interspaced short palindromic repeats (CRISPR) system is derived from a microbial adaptive immune system using a combination of a nuclease and a short RNA. Since its discovery in 1987 (109), CRISPR has been redesigned for a number of different gene-editing applications (4, 143, 144, 147, 148). In contrast to the nucleases mentioned above, for which specificity is dependent on protein–DNA interactions, the specificity of the CRISPR system relates to complementary RNA–DNA base pairing. This is “guided” by a single guide RNA’ (sgRNA) that contains a 20-nucleotide region designed to be complementary to the genomic DNA target termed the protospacer. Research has shown that partial mispairing is tolerated with the 3’ end of this 20 nucleotides being the most crucial (48, 149–151). It has been thought that this may increase the likelihood of off-target cleavage. Indeed, the level of off-target effects varies considerably among different targets, perhaps as a function of sgRNA design. The most commonly used CRISPR system today was derived from *Streptococcus pyogenes* and uses the nuclease Cas9. In contrast to ZFNs and TALENs, cleavage by Cas9 generates blunt DNA ends. CRISPR is 4.8 times more efficient at editing the CCR5 receptor than TALENs, as indicated by FACS followed by Sanger sequencing (152).

As with the other gene-editing strategies, CRISPR editing usually occurs in hematopoietic stem cells or in CD4+ T cells. In CD4+ T cells, CRISPR gene editing efficiency and engraftment has actually had worse efficiency than ZFNs. One of the first studies on CCR5 disruption using CRISPR in primary CD4+ T cells detected more than 30% of cells contained indels within the CCR5 gene using CRISPR/Cas9, compared to 40-60% using ZFNs (136). Furthermore, viral challenge with the R5-utilizing strain HIV-1_BaL_ and the transmitter/founder virus HIV-1_CH042_, individually, indicated that almost no p24 was produced from primary CD4+ T cells with a CCR5 knockdown at seven days post challenge, approximately 5 ng/ml, in contrast to control cells which demonstrated approximately 80 ng/ml of p24 expression. To validate specificity, the authors performed off-target analysis on the two gRNAs that were individually used in the study, specifically they amplified the 500 bp genomic regions spanning the top 15 sites with the most off-target potential for each gRNA. These amplified genomic regions were subjected to T7E1 analysis, wherein there was no amount of significant cleavage events detected, thus the authors concluded that no off-target effects occurred from these two gRNAs (153). A similar, more recent study using two distinct gRNAs in the CRISPR Cas9 system targeted toward the flanking regions of the CCR5Δ32 mutation locus estimated that less than 11% of primary CD4+ T cells were modified with the homozygous Δ32 mutation. Those that were modified showed almost no expression of p24 six days post HIV-1 challenge. No significant off-target effects were detected by whole genome sequencing (154). More recently, the CRISPR/AsCpf1 system, which is designed for easier multiplexing of gRNAs, multiplexed two gRNAs to knockdown the CCR5 receptor in primary CD4+ T cells *in vitro*. While a lentivirus was insufficient for transfection, an adenovirus achieved up to 28% disruption of CCR5, as determined by T7E1 assay. A p24 ELISA determined the p24 level post-14 days challenge with the CCR5-utilizing HIV-1_YU-2_ to be less than half of the control. As with previous experiments, no off-target effects were observed, and CCR5 disruption had no cytotoxic effects (155).

Studies in mice have fared about as well for CRISPR as they have for ZFNs. A study by Xu et al. demonstrated efficient knockdown of CCR5 in HSPCs which led to prevention of CCR5-utilizing HIV-1 infection when engrafted in mice (156). On average 32% of HSPCs were negative for CCR5, and an average of 8% of HSPCs successfully engrafted onto mice. Further, secondary transplantation of bone marrow cells from these mice onto naïve mice yielded 27% of CD4+ T cells with the CCR5 knockdown. The result of this CCR5 deletion in secondary transplanted mice prevented infection with CCR5-utilizing HIV-1_BaL-1_ infection eight weeks post infection that reduced HIV-1 RNA levels to almost half that of the control (156).

The most relevant example of CRISPR disrupting CCR5 is by Xu et al. which describes a patient that was diagnosed with HIV-1 infection and acute lymphoblastic leukemia in 2016, and immediately underwent ART and standard chemotherapy to treat these disorders (157). Later, in an attempt to cure both disorders, the patient received myeloablative conditioning, using cyclophosphamide and total-body radiation, and an allogeneic hematopoietic stem-cell transplant. The CRISPR/Cas9 system was used to disrupt the CCR5 receptor in HSPCs from a fully matched HLA donor and then transplanted into the patient. Between 5.2-8.28% of circulating bone marrow cells were found to have the CCR5 disruption, and whole genome sequencing of edited cells compared potential off-target sites in the genome of cells to the two gRNAs used, resulting in no DNA cleavage detected at any of these potential sites, thus the authors conclude that no off-target effects occurred after genome editing and at 19 months post-transplantation (157). While at 19 months post-transplantation the acute lymphoblastic leukemia was in full remission, the rapid viral rebound in response to ART interruption at 7 months post-transplantation indicates that this strategy does not successfully cure HIV-1 infection (157).

Cardozo-Ojeda et al. developed a mathematical model that projects the minimum threshold of CCR5 edited cells necessary to achieve a functional cure for HIV-1 infection. It was concluded that two criteria must be met to achieve a functional cure. First, the HPSCs transplanted into a patient must be five times more prevalent than endogenous HPSCs subsequent to total body radiation. The second criterion was that the frequency of transplanted HSPCs homozygous for the CCR5Δ32 allele in a patient must reach 76-94% (158). This model corroborates the ineffective strategy described by Xu et al. whose patient does not meet these criteria.

## 5 Combinatorial Approaches

Considering several factors are involved in the persistence of HIV-1 infection, including the establishment of latent viral reservoirs, it can be expected that a combinatorial approach will be necessary to achieve at least effective ART-independent control of HIV-1 infection. In addition to ATI with ART along with an investigational CCR5 therapy approach reviewed in Section 4, several novel combinatorial strategies that include inhibition of the CCR5 co-receptor as part of the approach are being investigated. These therapies aim to maximize the prevention of newly infected cells, to allow a better and safer outcome for HIV-1-infected patients that would eliminate the need for continuous ART. Additionally, they would provide the same advantages as coupling ART with CCR5 targeting, which is prevention of the emergence of CXCR4-utilizing virus and development of mutations that render CCR5-utilizing viruses still capable of entry.

The majority of novel therapeutic strategies involving CCR5 targeting combine this approach with CXCR4 inhibition (**Table 2**). This combinatorial approach may more completely prevent infection with the variety of HIV-1 strains within a patient, as well as prevent selective pressure on CXCR4-utilizing viruses. One such approach combined a modified form of RANTES (amino-oxypentane RANTES (AOP-RANTES)) to antagonize CCR5, and a modified Stroma-derived factor 1 beta (SDF-1β) with an added methionine (Met-SDF-1β) to antagonize CXCR4 (159). This study demonstrated that alone, each of these modified forms of the natural ligands RANTES or SDF-1β bound more efficiently to their responding co-receptor yet were suboptimal in inhibiting clinical HIV-1 isolates in PBMCs. However, when combined, their inhibition of infection with the isolates increased to 99% (159). This is just one approach to prevention of HIV-1 replication that involves co-receptor targeting, but which supports that drugs that are suboptimal on their own can have efficient additive or synergistic properties that may be more beneficial in the clinic.

**Table 2:** Combinatorial approaches utilizing CCR5 targeting techniques for therapy of HIV-1 infection.

Combination Approach	Methods		Study Stage	Model	Outcome
Inhibition of CCR5 and CXCR4	CCR5 inhibition with a modified form of RANTES, aminooxypentane (AOP)-RANTES, and CXCR4 inhibition with Stroma-derived factor 1 beta (SDF-1beta) derivative, Met-SDF-1beta.		*Ex vivo*	PBMCs	Combinations of these compounds inhibited mixed infections with R5 and X4 viruses (95 to 99%), whereas single drugs were less inhibitory (32 to 61%) (159)
	Dual CCR5/CXCR4 Antagonists	AMD3451	*In vitro and ex vivo*	PBMCs, monocytes, and macrophages	AMD3451 inhibited infection with clinical HIV-1 isolates or a variety of R5, R5/X4, and X4 strains of HIV-1 and HIV-2 at an IC_50_ ranging from 1.2 to 26.5 μM in various T cell lines, CCR5- or CXCR4-transfected cells, PBMCs, and monocytes/macrophages. (160)
		Ingenol derivatives	*In vitro and ex vivo*	MT-4 cells and PBMCs	Ingeol derivatives activated the HIV-1 LTR in MT-4 cells and primary CD4+ T cells with latent virus at 10 nM treatment, inhibited replication of HIV-1 subtuype B and C in MT-4 cells and PBMCs at EC_50_ of 0.02 and 0.09 μM, respectively, and induced downregulation of CD4, CCR5, and CXCR4 (80)
		Cumarin-based ligand GUT-70	*In vitro*	M1-CCR5 T cells	GUT-70 stabilized plasma membrane fluidity, inhibited HIV-1 entry, and down-regulated the expression of CD4, CCR5, and CXCR4. GUT-70 also inhibited HIV-1 replication through the inhibition of NF-κB (161)
		Suramin analog NF279	*Ex vivo*	MDMs infected with pseudoviruses	NF279 suppressed fusion of HIV-1 with MDMs, inhibited Ca^2+^ influx induced by R5 and X4 agonists, and antagonized gp120 mediated activation of CXCR4 (162)
		Pyrazolo-Piperidines	*In vitro*	PBMCs	Different compounds showed IC_50_ values ranging from 0.8 to 25 μM against R5 or X4 HIV-1 strains (163)
		Penicillixanthone A	*In vitro*	TZM-bl cells	Penicillixanthone A inhibited R5 and X4 HIV-1 at an IC_50_ of 0.36 and 0.20, respectively, but had moderate toxicity at 20.6 μM against TZM-bl cells (164)
Gene therapy targeting CCR5 and a suicide gene	Two-vector system: An integrating lentiviral vector expressing an HIV-1 Tat dependent TK-SR3 and a non-integrating lentiviral (NIL) vector expressing CCR5gRNA-CRISPR/Cas9 and HIV-1 Tat protein.		*In vitro*	TZM-bl cells	TZM-bl cells were stably integrated with TK-SR39 and were resistant to R5 HIV-1 (165)
Gene therapy targeting CCR5 in combination with a fusion inhibitor	Cal-1 comprising a short hairpin RNA to CCR5 (sh5) and a peptide that inhibits viral fusion with the cell membrane (C46)		*Ex vivo*	PBMCs	Cal-1 reduced CCR5 expression in PBMCs to CCR5Δ32 heterozygote levels and suppressed virus up to day 12. No escape mutations were present through 9 weeks of challenge. Cal-1 suppressed infection by different R5 viruses and inhibited virion internalization by 70% compared to 13% for C46 (166)

TK-SR39, Thymidine Kinase mutant SR39; LTR, Long Terminal Repeat; MDMs, primary human macrophages (monocyte-derived human macrophages).

Following this study, it was discovered primarily through other *in vitro* or *ex vivo* studies that antagonism of both CXCR4 and CCR5 was possible using the same compound and several such compounds have been identified (167). These dual antagonists vary greatly in structure and include peptide-based antagonists, pyrazole-based antagonists, bicyclams, and even naturally occurring compounds such as derivatives of ingenol or diterpene and other plant-derived compounds initially intended for treatment of other diseases. Among these, the N-pyridinylmethyl cyclam analog AMD is one of the first bicyclams to be discovered with dual CXCR4 and CCR5 antiviral properties (160). Princen et al. demonstrated this compound can efficiently inhibit infection of a variety of HIV-1 and HIV-2 isolates in various cell lines as well as primary cells with minimal toxicity to these cells, but no clinical study has followed since then (160, 167). Nonetheless, several other studies followed which demonstrated other dual antagonists can successfully inhibit various HIV-1 strains *in vitro* or *ex vivo* while exhibiting low toxicity (80, 161–164, 168).

A decade later in 2014, Abreu et al. demonstrated that ingenol derivatives (ISDs) isolated from *Euphorbia tirucalli* can likewise inhibit X4 and R5 viruses *in vitro* and *ex vivo*. Treatment of PBMCs and MT-4 human T cells with ISDs was shown to inhibit HIV-1 subtype B and C replication at comparable EC_50_s to drugs used in ART (80). Interestingly, this study also demonstrated potential latency reactivation properties of ISDs. When different reporter cell lines and infected CD4+ T cells from five ART-suppressed patients were treated with ISDs, LTR activation was induced (80). The results of this study provide hope that LTR-driven transcription to reactivate HIV-1 in latently infected cells, along with prevention of HIV-1 infection through downregulation of co-receptors required for entry can both be accomplished using the same modality (80).

Shortly thereafter, other natural products were shown to be dual co-receptor antagonists. GUT-70, a natural product derived from *Calophyllum brasiliense*, was shown to down-regulate the expression of CD4, CCR5, and CXCR4 in M1-CCR5 cells and inhibit entry of X4 and R5 viruses. While the downregulation of these receptors was significantly correlated with reduced infectivity, other mechanisms of GUT-70 action were discovered to greatly contribute to its antiviral effect. These include reducing the membrane fluidity of cells to disrupt viral entry as well as inhibition of NF-κB to prevent viral replication (161, 169). Considering these various cellular components modulated by GUT-70 are necessary for broader biological processes as compared to the inhibition of the co-receptors alone, further understanding of these antiviral mechanisms is necessary to predict potential off-target effects of GUT-70 in HIV-1-infected patients. Moreover, GUT-70 has also previously demonstrated anti-leukemic properties (170). Dual co-receptor antagonism using this compound for treatment of HIV-1 infection can thus provide the additional advantage of simultaneous prevention or treatment of lymphoma or leukemia, which overcomes potential toxicity of drug-drug interactions normally associated with anti-tumor agents and ART (161).

Accordingly, re-evaluation of the antiviral mechanism of even previously characterized anti-HIV-1 compounds has revealed that they also prevent HIV-1 infection through dual co-receptor antagonism. The NF279 was initially reported as an HIV-1 fusion inhibitor that prevents HIV-1 infection by blocking P2X1 channels (171). A recent study, however, demonstrated that it does not inhibit HIV-1 fusion by preventing the activation of P2X1 channels, but by antagonizing CXCR4 and CCR5 signaling through suppression of Ca^2+^ responses in primary macrophages induced by gp120 binding (162). This recent investigation on NF275 is one of the few current studies evaluating the antiviral mechanism of these novel compounds beyond their co-receptor binding properties. Another study evaluated the mechanism of a compound containing pyrazole-piperidine core, which was originally identified through a GPCR-guided screen (163). This compound was found to prevent HIV-1 entry with X4 or R5 strains, but primarily due to its non-nucleoside reverse transcriptase (NNRT) activity as opposed to its co-receptor antagonistic properties (163). While the predominant mode of action of this compound was identified to be its inhibition of HIV-1 RT, its additional dual chemokine antagonism was proposed to delay development of HIV-1 resistance when compared to other NNRTIs (163). Therefore, a novel compound may not strongly inhibit binding of HIV-1 to CXCR4 or CCR5, but may still exert robust antiviral efficacy and protect against infection through additional predominant or complementary mechanisms.

Another compound investigated, penicillixanthone A (PXA), is a natural xanthone dimer derived from the fungus *Aspergillus fumigates* that also exerts dual co-receptor antagonistic effects. This dimer was described to have potent anti-HIV-1 activity due to inhibition of infection with R5-tropic HIV-1 SF162 and CXCR4-tropic HIV-1 NL4-3 in TZM-bl cells, with an IC_50_ of 0.36 and 0.26 μM, respectively (164). However, it exhibited moderate toxicity in TZM-bl cells and thus has a major disadvantage as compared to other dual co-receptor antagonists currently under investigation (164).

While dual targeting of CCR5 and CXCR4 co-receptors appears promising for control of HIV-1 infection, there are concerns that ablating the CXCR4 receptor in certain cell types will lead to detrimental effects. This is likely due to the important role of CXCR4 in maintaining normal function of hematopoietic stem cells (172, 173). In mice, for example, CXCR4 deficiency causes embryonic lethality or malignancy (174, 175). This highlights the persistent challenges faced with development of effective combinatorial approaches—that is, maintaining high antiviral efficacy with low risk of adverse effects in the HIV-1-infected patient.

In addition to CXCR4 inhibition, another combinatorial approach investigated shRNA targeting of CCR5 in combination with the fusion inhibitor C46, a gp41-derived C peptide (166, 176). Not only was this approach demonstrated to be effective against different strains of HIV-1, but mutant viruses were also not detected in infected PBMCs over a week later (166, 176). While the rise of mutants or CXCR4 tropic viruses can still occur months to years later, these recent studies indicate that more assays are being incorporated to assess novel drug or therapy efficacy at the *in vitro* stage to better avoid this outcome.

Alternatively, emergence of CXCR4-utilizing viruses can be avoided using a combinatorial approach that utilizes an HIV-1 protein-dependent suicide gene. This was accomplished by introducing a CCR5 gRNA-CRISPR/Cas9 system into TZM-bl cells to knockout CCR5 along with an HIV-1 Tat-dependent suicide gene TK-S39 (165). This novel approach allows expression of HIV-1 Tat, which can occur when a CXCR4-utilizng or CCR5 antagonist-resistant virus enters and replicates in a cell, to induce cell death and prevent cell-to-cell spread of HIV-1 in the occurrence of HIV-1 replication despite CCR5 knockdown. The introduction of a suicide gene has in fact been previously argued to be a necessity for CCR5 therapy, which otherwise would fail due to expansion of CXCR4-utilizing viruses and selection of CCR5 antagonist-resistant strains among other factors (177). How clinical efficacy of this or other combinatorial approaches discussed would compare with a more established preventative method like ART adherence during CCR5 therapy has yet to be assessed.

## 6 Discussion

Several therapeutic strategies targeting CCR5, either through blockade of the co-receptor or through gene editing techniques to inhibit its expression, have demonstrated the potential of CCR5 ablation to inhibit HIV-1 infection, at least temporarily. Accordingly, FDA-approved CCR5 targeting therapeutics such as Miraviroc can be used for patients for which ART may not be suitable (110).

As with all HIV-1 therapeutic strategies being investigated, ART-independent control of HIV-1 infection through CCR5 targeting is promising but major hurdles persist for the development of a cure. Common characteristics of HIV-1 infection such as establishment of latently infected reservoirs, impracticality of therapy delivery to anatomically privileged sites, and the ongoing development of drug resistant viruses continue to challenge efficacies of CCR5 targeting strategies (178). Furthermore, even after optimization of delivery of gene editing tools or of the potency of CCR5 antagonists, there is insufficient data to support that the majority of HIV-1 susceptible cells in a patient can be targeted. This then suggests inhibition of viral replication will still be necessary, which currently is only feasibly accomplished with ongoing ART adherence. Therefore, additional clinical data is needed to support that the therapeutic outcome of individuals undergoing CCR5 targeting therapy will be a functional cure similar to that which occurred for the Berlin or London patients. Instead, CCR5 inhibition alone may serve a more supplementary approach to prevent disease progression and compensate for the shortcomings of ART.

To overcome these limitations of CCR5 monotherapies, many combinatorial approaches have been investigated in recent years and show potential for more efficient inhibition of viral infection with diverse HIV-1 strains as well as avoidance or delay of the development of resistant strains (179). However, in comparison to the vast research on combinatorial methods for HIV-1 therapy or cure, integration of CCR5 targeting appears to be at the beginning *in vitro* stages. Considering these approaches target other cellular functions which may be detrimental to patients, clinical data assessing the safety of these approaches is needed. If evaluated to be safe in patients, many of these approaches including the use of dual co-receptor antagonists, will demonstrate that inhibition of HIV-1 infection without the requirement of life-long ART adherence will at least be feasible.

## Author Contributions

HM, TG, RB, and MC, conceptualization, writing-original draft, editing, and visualization. IS, writing-review, editing, and funding acquisition. MN and BW, conceptualization, writing-review and editing, visualization, supervision, project administration, and funding acquisition. All authors contributed to the article and approved the submitted version.

## Funding

These studies were funded in part by the Public Health Service, National Institutes of Health, through grants from the National Institute of Mental Health (NIMH) R01 MH110360 (Contact PI, BW), the NIMH Comprehensive NeuroAIDS Center (CNAC) P30 MH092177 (KK, PI; BW, PI of the Drexel subcontract involving the Clinical and Translational Research Support Core), National Institute of Allergy and Infectious Disease (NIAID) Martin Delaney Collaboratories for HIV Cure Research (UM1 AI164568 (contact PI KK, Multi-PI TB; BW, PI of the Drexel subcontract to RF3; MN, PI of Drexel subcontract to RF1/RF3), and under the Ruth L. Kirschstein National Research Service Award T32 MH079785 (BW, Principal Investigator of the Drexel University College of Medicine component. The contents of the paper are solely the responsibility of the authors and do not necessarily represent the official views of the NIH.

## Conflict of Interest

The authors declare that the research was conducted in the absence of any commercial or financial relationships that could be construed as a potential conflict of interest.

## Publisher’s Note

All claims expressed in this article are solely those of the authors and do not necessarily represent those of their affiliated organizations, or those of the publisher, the editors and the reviewers. Any product that may be evaluated in this article, or claim that may be made by its manufacturer, is not guaranteed or endorsed by the publisher.
